# 脉冲直流电喷雾电离质谱快速检测生活污水中的11种常见毒品

**DOI:** 10.3724/SP.J.1123.2025.01031

**Published:** 2025-08-08

**Authors:** Qiaocui SHI, Shengjun CHEN, Jiayu FU, Weihong XIE

**Affiliations:** 1.浙江警察学院，浙江省毒品防控技术研究重点实验室，浙江 杭州 310053; 1. Key Laboratory of Drug Prevention and Control Technology of Zhejiang Province，Zhejiang Police College，Hangzhou 310053，China; 2.浙江省检验检疫科学技术研究院，浙江 杭州 311202; 2. Zhejiang Institute of Inspection and Quarantine Science and Technology，Hangzhou 311202，China; 3.杭州市公安局刑事科学技术研究所，浙江 杭州 310004; 3. Institute of Criminal Science and Technology，Hangzhou Municipal Public Security Bureau，Hangzhou 310004，China

**Keywords:** 脉冲直流电喷雾电离质谱, 常见毒品, 生活污水, 快速检测, pulsed direct current electrospray ionization mass spectrometry（pulsed-DC-ESI-MS）, common drugs, domestic sewage, rapid detection

## Abstract

针对现有污水样本快速检测技术存在的不足，以及传统实验室质谱技术无法应用于现场快速检测、检测耗时较长等问题，本文提出了一种基于脉冲直流电喷雾电离质谱（pulsed-DC-ESI-MS）测定生活污水中11种常见毒品（吗啡、甲基苯丙胺、去甲氯胺酮、苯丙胺、3，4-亚甲基双氧甲基苯丙胺、可卡因、6-单乙酰吗啡、3，4-亚甲基双氧苯丙胺、苯甲酰爱康宁、氯胺酮、可待因）的快速检测方法。污水样品经浓盐酸调节pH至2，用Oasis PRiME MCX固相萃取柱进行萃取，萃取液经氮吹至近干后用200 μL甲醇复溶，涡旋0.5 min；复溶后的样品溶液经0.22 μm有机相滤膜过滤后采用pulsed-DC-ESI-MS进行分析。方法学验证结果表明，11种毒品在各自的线性范围内具有良好的线性关系，相关系数（*r*^2^）均≥0.998 6，检出限（LOD）为0.01~0.5 μg/L，定量限（LOQ）为0.05~5 μg/L。在低、中、高3个加标水平下，11种毒品的回收率为88.0%~107.6%，日内和日间精密度均≤8.5%。该方法检测速度快，大大提高了检测效率，适用于生活污水中常见毒品的快速检测分析。

## Abstract (en)

Drugs and their metabolites are ubiquitous in various environmental media， including water bodies， soil， sediment， and air. These substances pose significant risks to human and animal safety and have emerged as a new category of pollutants. Accurate detection of the content of drugs in the environmental matrix can not only timely and objectively grasp the usage status， spatial distribution characteristics and temporal change patterns of drugs in a certain area， but also precisely assess the impact of long-term low-level exposure on the ecological environment. Currently， monitoring domestic sewage has emerged as a significant tool for tracking drugs use in various countries globally. Data obtained from domestic sewage samples for drugs detection can provide an objective， real-time， accurate， and effective reflection of local drug use patterns. Mass spectrometry has the advantages such as high specificity， high sensitivity and high quantitation accuracy and is mostly used for the analysis of domestic sewage samples. However， laboratory mass spectrometry methods involve complicated and time-consuming sample preparation processes， along with stringent environmental requirements， making them impractical for on-site rapid analysis. Consequently， there is an immediate need for the development of detection methods that are broad-spectrum， rapid， and capable of high throughput. In recent years， direct ionization mass spectrometry technology has been widely applied in on-site rapid detection and analysis work due to its characteristics such as avoiding complex chromatographic separation processes， fast analysis speed， high matrix tolerance， the ability to directly ionize analytes in complex matrix samples， and no（or only very little） sample pretreatment required. Direct ionization mass spectrometry is now playing an essential role in scientific research， environmental monitoring and public security. Pulsed direct current electrospray ionization mass spectrometry（pulsed-DC-ESI-MS） is a portable detection platform， which can be used for the accurate， rapid， on-site， batch， and sensitive analysis of picoliter level samples. In this research， a novelty method of pulsed-DC-ESI-MS was developed to achieve rapid detection of 11 drugs in domestic sewage. The drugs analyzed include methamphetamine（MA）， amphetamine（AM）， 3，4-methylenedioxymethylamphetamine（MDMA）， 3，4-methylenedioxyamphetamine（MDA）， morphine（MOR）， 6-monoacetylmorphine（6-MAM）， ketamine（KET）， norketamine（NKET） cocaine（COC）， benzoylecgonine（BZE）， and codeine（COD）. The sewage samples were extracted using the Oasis PRiME MCX solid-phase extraction（SPE） column. The extraction solution was blowing to nearly dry with nitrogen and then redissolved with 200 μL methanol and vortex for 0.5 min. The redissolved sample solution was filtered through a 0.22 μm organic filter membrane and analyzed by pulsed-DC-ESI-MS. The methodological validation results indicated that the 11 drugs had a good linear relationship within their respective linear ranges. The correlation coefficients（*r*^2^） were all ≥0.998 6， the limits of detection（LODs） and quantification（LOQs） were 0.01‒0.5 μg/L and 0.05‒5 μg/L， respectively. At three spiked levels， the recoveries of 11 drugs ranged from 88.0% to 107.6%， the intra- and inter-day precisions were both ≤8.5%. This method has a fast detection speed， greatly improving the detection efficiency， and is suitable for the rapid detection and analysis of common drugs in domestic sewage.

毒品及管制药物的滥用会严重威胁人类健康与社会稳定。随着毒品及管制药物制造、贩运和滥用活动的不断发生，其母体及其代谢产物会持续进入大气、水体、土壤等环境介质，进而成为新兴污染物，对生态环境造成不可逆转的严重损害^［1‒3］^。准确检测毒品及管制药物在环境基质中的含量，不仅能够及时、客观地掌握某地区毒品及管制药物的使用状况、空间分布特征以及时间变化规律，还能精准评估长期低水平暴露对生态环境造成的影响^［4］^。毒品和管制药物以及它们的代谢物最容易存在的环境基质是水体^［5］^。当前，针对水体中毒品及管制药物的分析，大多采用固相萃取（SPE）与液相色谱-串联质谱（LC-MS/MS）联用技术^［6‒10］^。Bijlsma等^［11］^从城市污水处理厂采集样品后，利用SPE法进行前处理，随后采用LC-MS/MS技术对样品展开分析，并基于城市污水分析结果推算了哥伦比亚主要城市的毒品使用状况。李喜青课题组^［12‒14］^借助LC-MS/MS技术，在国内多个大城市开展了基于水体中毒品和管制药物的监测研究。

近年来，直接电离质谱技术凭借其可避免复杂色谱分离过程、分析速度快、基质耐受性高、能够直接电离复杂基质样品中的分析物且无需（或仅需极少量）样品预处理等特性，被广泛应用于现场快速检测与分析工作中^［15‒17］^。Fabregat等^［18］^利用大气固体分析探针（ASAP）与质谱技术，实现了对不同类型样品及其表面新精神活性物质的快速筛选。Silva等^［19］^采用纸喷雾质谱技术，仅需60多秒便能完成对190多种芬太尼类物质的检测分析。脉冲直流电喷雾电离质谱（pulsed-DC-ESI-MS）是一种集成了pulsed-DC-ESI技术与小型MS系统的便携式快速检测平台，能够对多种物质进行现场迅速检测与分析^［20］^。与其他直接电离技术相比，pulsed-DC-ESI-MS凭借其体积小、重量轻且无需载气的优势，在现场快速检测分析领域展现出广阔的应用前景^［21］^。本课题组^［22］^利用pulsed-DC-ESI-MS技术，实现了对尿液和毛发中苯丙胺类物质的现场快速检测与分析。此外，基于pulsed-DC-ESI-MS技术，本课题组^［23］^还建立了一种现场快速检测尿液中28种芬太尼类物质的实验方法，检出限（LOD）低至0.001~0.5 ng/mL。

本研究基于pulsed-DC-ESI-MS技术，通过优化前处理条件与质谱参数，建立了一种针对生活污水中11种常见毒品的现场快速检测技术。该检测技术能够有效克服传统检测方法前处理过程复杂、耗时较长、成本较高等弊端，可实现污水样品的现场采样、进样及原位电离分析，从而满足批量样品现场快速分析的需求。

## 1 实验部分

### 1.1 仪器、试剂与材料

AMS-100便携式脉冲直流电喷雾电离质谱仪、w50一次性现场快速检测探针（宁波华仪宁创智能科技有限公司）；Sorvall ST 16离心机、LabTower EDI 15超纯水仪（美国Thermo Fisher公司）；MSI涡旋仪（德国IKA公司）；MD200-1氮吹仪（杭州奥盛仪器有限公司）；Oasis PRiME MCX固相萃取柱（3 mL/60 mg）、Oasis PRiME HLB固相萃取柱（3 mL/60 mg）、Oasis WCX固相萃取柱（3 mL/60 mg）、WAT200609固相萃取仪（美国Waters公司）； 2 mL聚丙烯离心管（美国KIRGEN公司）。

11种毒品标准品（质量浓度均为1 mg/mL，溶剂为甲醇）：甲基苯丙胺（MA）、苯丙胺（AM）、3，4-亚甲基双氧甲基苯丙胺（MDMA）、3，4-亚甲基双氧苯丙胺（MDA）、吗啡（MOR）、6-单乙酰吗啡（6-MAM）、氯胺酮（KET）、去甲氯胺酮（NKET）、可卡因（COC）、苯甲酰爱康宁（BZE）、可待因（COD）（上海原思标物科技有限公司）。11种相应的氘代同位素内标（质量浓度均为100 μg/mL，溶剂为甲醇）：D5-MA、D5-AM、D4-MDMA、D4-MDA、D3-MOR、D3-6-MAM、D4-KET、D3-NKET、D3-COC、D3-BZE、D3-COD（公安部第三研究所）。以上标准品溶液均置于‒80 ℃冰箱中避光保存备用。乙腈、甲醇、乙醇（色谱级，德国Merck公司）；甲酸（色谱级，上海阿拉丁生化科技有限公司）；浓盐酸、浓氨水（分析纯、北京化学工业集团有限责任公司）。

实际生活污水样品分别采集自浙江省的6个污水处理厂，样品采集后置于聚对苯二甲酸乙二醇酯材质的塑料瓶中，于‒80 ℃下密封储存。

### 1.2 标准溶液的配制

混合标准溶液的配制：分别取1 mg/mL的11种毒品标准品溶液（各10 μL）和0.1 %甲酸甲醇溶液（890 μL）于2 mL离心管中，涡旋30 s，配制成质量浓度为10 mg/L的11种毒品混合标准溶液，于‒40 ℃下保存。

混合内标溶液的配制：分别取100 μg/mL的11种氘代同位素内标溶液（各10 μL）和0.1 %甲酸甲醇溶液（890 μL）于2 mL离心管中，涡旋30 s，配制成质量浓度为1 mg/L混合内标溶液，于‒40 ℃下保存。

### 1.3 样品前处理

将‒80 ℃下的污水样品置于4 ℃下解冻，放至室温并充分摇匀。移取50 mL污水样品于具塞瓶中，加入浓盐酸调节pH至2，再加入100 μL混合内标溶液，抽滤后再用Oasis PRiME MCX固相萃取柱进行萃取。首先分别用4 mL甲醇和4 mL超纯水对Oasis PRiME MCX固相萃取柱进行活化，随后将50 mL样品上样过柱；萃取完成后，用4 mL甲醇对固相萃取柱进行淋洗，以去除部分杂质及干扰物。用10%氨水甲醇溶液进行洗脱，收集洗脱液，在20 ℃下氮吹至近干，用200 μL甲醇复溶，再涡旋0.5 min，经0.22 μm有机相针孔滤膜过滤后进行pulsed-DC-ESI-MS分析。需强调的是，整个前处理过程中的上样流速均不超过10 mL/min。

### 1.4 仪器条件

扫描模式：正离子扫描；电喷雾电压：3 kV；传输毛细管温度：275 ℃；传输毛细管电压：35 V；离子提取透镜电压：110 V；碰撞气：高纯氦气；质量扫描范围：40~500 Da。11种分析物的质谱参数见表1。

**表1:** 11种目标分析物及同位素内标的质谱参数

Analyte	*M*_r_/Da	Parent ion（*m/z*）	Fragment ions（*m/z*）	Fragmentation voltage/V
Methamphetamine（MA）	149.23	150.08	119.00^*^， 91.00	2.5
3，4-Methylene-dioxymethylamphetamine（MDMA）	193.24	194.14	163.08^*^， 58.15	3.0
3，4-Methylene-dioxyamphetamine（MDA）	179.22	180.29	163.06^*^， 149.00	2.0
Amphetamine（AM）	135.21	136.19	119.00^*^， 91.00	2.0
Ketamine（KET）	237.72	238.10	220.10^*^， 207.10	2.0
Morphine（MOR）	285.30	286.20	201.00^*^， 211.00	2.5
Codeine（COD）	299.40	300.30	215.20^*^， 183.10	3.0
6-Monoacetylmorphine（6-MAM）	327.40	328.10	193.10， 211.10^*^	3.0
Cocaine（COC）	303.35	304.30	182.10^*^， 150.00	2.0
Norketamine（NKET）	223.70	224.20	207.00^*^， 125.10	2.5
Benzoylecgonine（BZE）	289.33	290.13	105.12， 168.11^*^	2.5
D5-MA	154.23	155.08	121.00^*^	2.5
D4-MDMA	233.70	198.14	167.07^*^	2.0
D4-MDA	183.22	184.20	167.11^*^	2.0
D5-AM	140.21	141.20	124.10^*^	2.0
D4-KET	241.75	242.00	224.00^*^	3.5
D3-MOR	288.30	289.20	211.00^*^	2.5
D3-COD	302.40	303.10	215.10^*^	3.0
D3-6-MAM	330.40	331.00	211.00^*^	3.0
D3-COC	306.37	307.00	185.00^*^	2.5
D4-NKET	227.68	228.20	211.10^*^	3.0
D3-BZE	292.33	293.13	171.12^*^	2.5

* Quantitative ion.

## 2 结果与讨论

### 2.1 质谱参数优化

蘸取适量100 μg/L的混合标准溶液，注入pulsed-DC-ESI-MS进行快速检测。在正离子全扫描模式下，采集并记录11种毒品的母离子信号。根据相对分子质量、［M+H］^+^的*m/z*以及全扫描模式下的峰形来确定母离子，相对应的子离子通过二级质谱扫描进行确定。选取响应值最高的子离子作为定量离子，将响应值次之的子离子作为定性离子，同时确定最佳碎裂电压。11种目标分析物的一级和二级质谱图见附图1（www.chrom-China.com）。11种分析物的质谱参数如表1所示。

### 2.2 样品前处理方法的优化

#### 2.2.1 固相萃取柱的选择

以11种目标分析物的回收率为考察指标，实验分别比较了PRiME HLB柱、WCX柱和PRiME MCX柱3种固相萃取柱的萃取效果。结果如图1所示，当使用MCX柱进行固相萃取时，11种目标分析物的回收率最高，且MCX柱比HLB柱和WCX柱有更好的选择性，基质干扰小。因此，本实验选用Oasis PRiME MCX固相萃取柱对污水样品进行前处理。

**图1:**
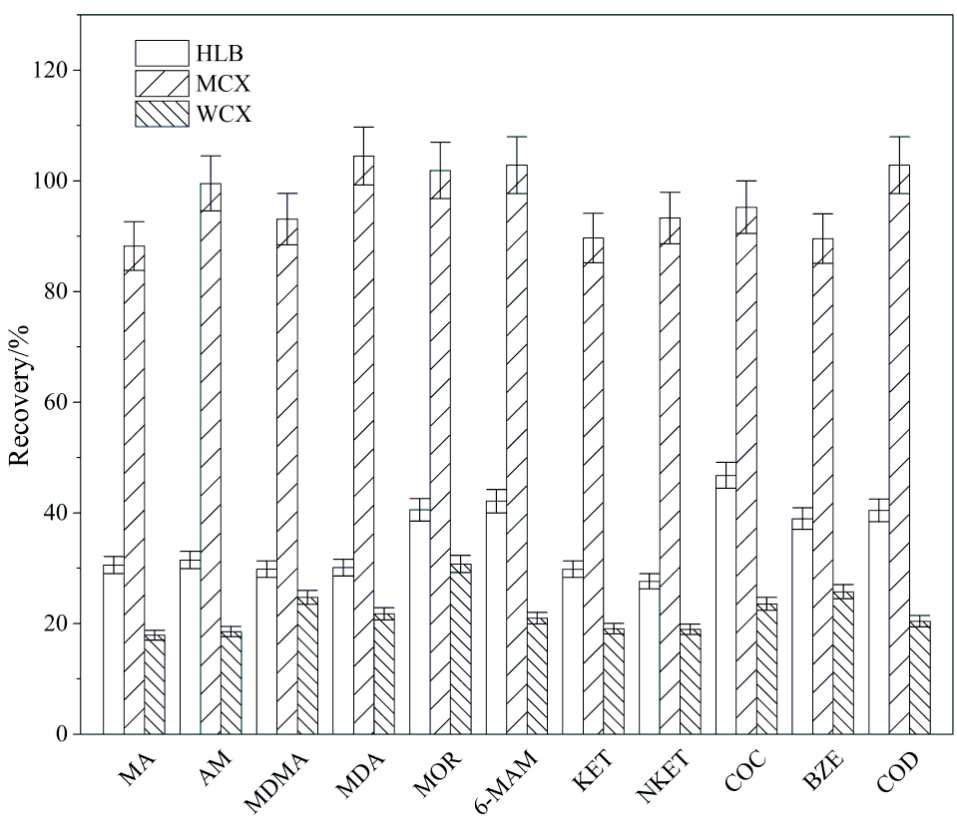
3种不同固相萃取柱对11种目标分析物回收率的影响（*n*=5）

#### 2.2.2 水样pH的优化

在使用MCX柱萃取目标分析物之前，需调节污水样品的pH值。当目标分析物经酸化处理后呈现阳离子特征时，基于电荷相互作用与分子间作用力，阳离子化合物可被选择性地保留在MCX柱上。以11种目标分析物的响应值为考察指标，通过比较11种分析物（50 μg/L）在不同pH（1、2、3、4）下的响应值来选取最优pH值。为确保实验结果的准确性和可靠性，每组实验均平行测定3次。结果如图2所示，除AM和COD外，其余分析物在水样pH为2时的响应值明显高于pH为1和3时的响应值。这可能是因为适宜的pH能使样品中各分析物的水解和电离程度基本达到最优状态，促使各物质尽可能充分地吸附在Oasis PRiME MCX固相萃取柱上，进而提高萃取效率。此外，当水样pH=4时，所有目标分析物均无法被检出。因此，本实验选择将污水样品的pH调节至2。

**图2:**
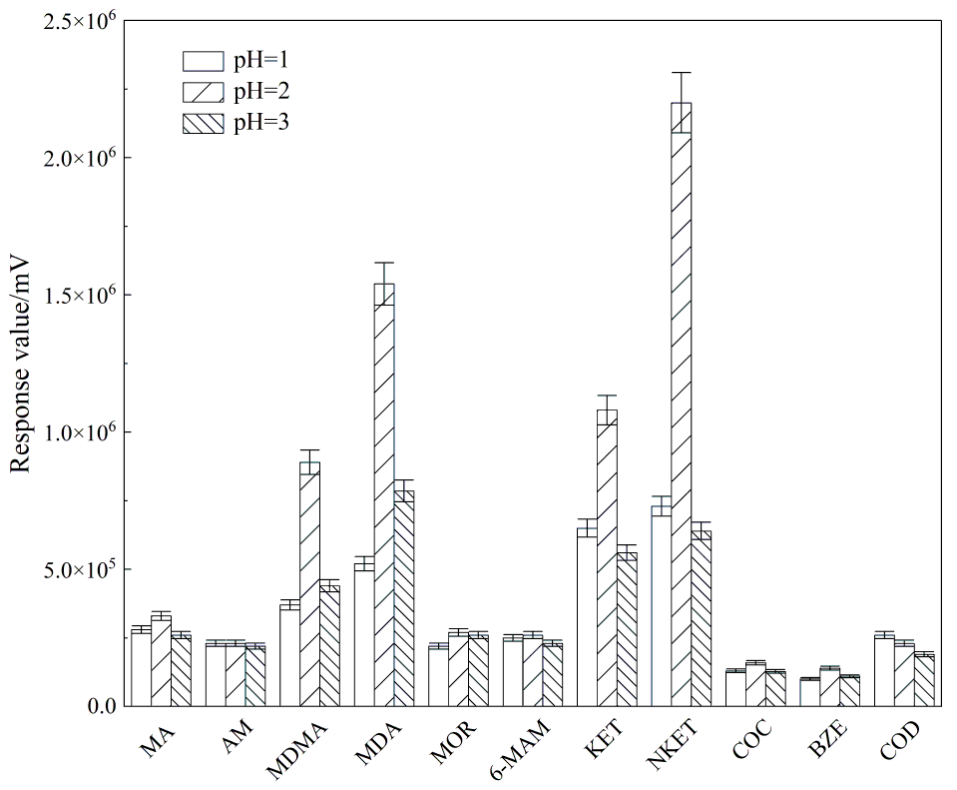
不同pH条件下11种目标分析物的响应值（*n*=3）

#### 2.2.3 洗脱液中氨水体积分数的优化

考察了洗脱液（氨水甲醇溶液）中不同体积分数（5%、10%、20%、40%、50%、60%、70%、80%）的氨水对目标分析物的洗脱效果。实验结果表明，大部分目标分析物在50%氨水甲醇溶液洗脱下达到了最高响应值，当氨水的体积分数超过50%后，大部分目标化合物的响应值开始降低；然而，除KET外，其余10种目标分析物的响应值在氨水体积分数为10%~50%的区间内增加得较为缓慢，考虑到实际污水样品中的杂质含量较高，若选用50%氨水甲醇溶液作为洗脱液，可能会增加共流出杂质的洗脱量，从而影响质谱分析。因此，本实验最终选择10%氨水甲醇溶液作为11种毒品的洗脱液。

### 2.3 方法学验证

#### 2.3.1 线性范围、检出限与定量限

在优化的实验条件下使用内标法进行定量分析。用0.1 %甲酸甲醇溶液配制系列质量浓度（0.05、0.1、0.5、1、2、5、10、20、50、100 μg/L）的混合标准溶液，经样品前处理后进样分析。以目标分析物的质量浓度为横坐标（*x，* μg/L），以目标分析物与对应内标定量离子的响应值比值为纵坐标（*y*），绘制标准曲线，得到线性回归方程。如表2所示，11种目标分析物在各自的线性范围内具有良好的线性关系，相关系数（*r* ^2^）均≥0.998 6。其中，COC的线性范围跨越了3个数量级，为保证其校准曲线的准确性，将该校准曲线划分为低浓度和高浓度两条曲线，两条曲线的斜率基本保持一致。11种目标分析物的标准曲线图见附图2。11种目标分析物的检出限（LOD）和定量限（LOQ）分别通过3倍和10倍信噪比（*S*/*N*）获得。根据实验结果计算得到11种目标分析物的LOD和LOQ分别为0.01~0.5 μg/L和0.05~5 μg/L。

**表2:** 11种目标分析物的线性方程、相关系数、线性范围、检出限和定量限

Analyte	Linear equation	*r* ^2^	Linear range/（μg/L）	LOD/（μg/L）	LOQ/（μg/L）
MA	*y*=0.2004*x‒*0.0086	0.9992	0.5‒50	0.1	0.5
MDMA	*y*=0.2192*x‒*0.0046	0.9988	0.5‒50	0.1	0.5
MDA	*y*=0.2111*x‒*0.0072	0.9998	0.5‒50	0.1	0.5
AM	*y*=0.1944*x*+0.0054	0.9986	0.5‒50	0.05	0.5
KET	*y*=0.2171*x‒*0.0016	0.9998	0.5‒50	0.1	0.5
NKET	*y*=0.2057*x*+0.0005	0.9998	0.5‒50	0.1	0.5
COC	*y*=2.8152*x*+0.0355	0.9994	0.05‒2	0.01	0.05
	*y*=2.7739*x‒*1.0047	0.9998	2‒50		
COD	*y*=0.1015*x‒*0.0031	0.9995	1.0‒100	0.2	1
MOR	*y*=0.2301*x‒*0.0127	0.9997	5.0‒100	0.5	5
6-MAM	*y*=0.0858*x‒*0.0025	0.9987	2.0‒100	0.5	2
BZE	*y*=0.2372*x‒*0.0027	0.9994	0.5‒50	0.1	0.5

*y*： the ratio of the response value of the target analyte to the corresponding internal standard； *x*： mass concentration， μg/L.

#### 2.3.2 回收率与精密度

为了验证方法的准确性和重复性，在空白污水样品中分别添加低、中、高3个水平的混合标准溶液，按1.3节和1.4节方法进行加标回收试验。每个加标水平样品平行制备5份，计算平均回收率；每个加标水平在1 d内平行测定5次，计算日内精密度（intra-day RSD）；连续测定6天，计算日间精密度（inter-day RSD）。如表3所示，在低、中、高3个加标水平下，11种目标分析物的回收率为88.0%~107.6%，日内和日间精密度均≤8.5%，说明所建方法的精密度良好，能够满足11种毒品的分析要求。

**表3:** 11种目标分析物的回收率、日内和日间精密度

Analyte	Spiked level/（μg/L）	Recovery/% （*n*=5）	Intra-day RSD/% （*n*=5）	Inter-day RSD/% （*n*=6）
MA	0.5	104.4	3.5	5.5
	5	102.3	4.9	5.8
	50	88.0	5.7	2.9
AM	0.5	104.4	7.2	5.9
	5	102.3	5.6	8.3
	50	99.5	6.0	5.2
MDMA	0.5	99.0	4.3	4.7
	5	97.2	7.4	5.8
	50	93.1	6.8	5.9
MDA	0.5	100.5	5.7	6.8
	5	99.4	6.1	5.1
	50	103.7	5.8	4.2
KET	0.5	105.5	4.6	3.0
	5	101.9	6.5	6.4
	50	92.7	5.5	7.1
NKET	0.5	101.6	7.5	8.3
	5	105.1	6.8	7.3
	50	97.3	7.0	5.5
MOR	5	95.3	5.9	7.7
	20	97.5	6.8	3.7
	100	101.9	5.7	4.2
6-MAM	2	103.8	7.3	6.3
	10	104.4	6.0	6.5
	100	102.9	5.7	6.3
COC	0.05	107.6	3.6	6.0
	1	90.3	6.8	3.6
	50	95.1	5.7	7.5
BZE	0.5	98.9	6.4	5.3
	5	102.4	5.3	3.8
	50	89.5	5.8	4.3
COD	1	103.8	4.3	2.9
	10	104.4	6.6	8.5
	100	102.9	7.0	5.2

### 2.4 实际生活污水样品的检测

采集浙江省6个污水处理厂的实际生活污水样品，按1.3节方法进行前处理，按1.4节方法进样分析。实验结果表明，在6个实际污水样品（S1~S6）中共检出MA、MOR、KET、COC、BZE及MDMA等6种常见毒品，检出水平为0.002 3~0.102 6 μg/L，其余5种毒品均未检出；并且，利用该方法完成单个污水样品中一种或多种毒品物质的检测仅需约20 s。为了验证本方法在实际样品检测中的准确性，将本方法检测结果与采用《法庭科学水样中吗啡等10种毒品及代谢物检验 液相色谱-质谱法》（GA/T 2059-2023）^［24］^得到的检测结果作比较。按照GA/T 2059-2023^［24］^设定HPLC-MS/MS的仪器条件，实验得到的检测结果如表4所示。采用配对样本*t*检验统计学方法对两种方法的检测结果进行分析，结果显示，*t*值为1.734 6，对应的*P*值为0.510 7，由于*t*>*P*，说明两种方法的检测结果无明显差异^［25］^。

**表4:** 两种方法在实际污水样品检测中的结果 (μg/L)

Analyte	HPLC-MS/MS							pulsed-DC-ESI-MS					
	S1	S2	S3	S4	S5	S6		S1	S2	S3	S4	S5	S6
MA	ND	ND	ND	0.0026	0.0537	ND		ND	ND	ND	0.0023	0.0506	ND
MOR	0.1237	ND	ND	0.0478	0.0314	ND		0.1026	ND	ND	0.0493	0.0297	ND
KET	ND	ND	0.0065	ND	ND	ND		ND	ND	0.0063	ND	ND	ND
COC	ND	ND	ND	ND	ND	0.0487		ND	ND	ND	ND	ND	0.0512
BZE	0.0061	0.0082	ND	ND	ND	ND		0.0057	0.0078	ND	ND	ND	ND
MDMA	ND	ND	ND	0.0546	ND	ND		ND	ND	ND	0.0617	ND	ND

pulsed-DC-ESI-MS： pulsed direct current electrospray ionization mass spectrometry； ND： not detected.

## 3 结论

本文基于pulsed-DC-ESI-MS技术，构建了一种快速检测生活污水中11种常见毒品的分析方法。该方法可规避复杂的色谱前处理流程，具备耗时短、溶剂用量少、高通量以及操作简便等优势，能够有效提升生活污水中常见毒品物质的检测效率。目前，该方法已成功应用于浙江省6个污水处理厂实际污水样品的检测分析，并取得了满意的结果。
